# Daily Leucine Intake Is Positively Associated with Lower Limb Skeletal Muscle Mass and Strength in the Elderly

**DOI:** 10.3390/nu13103536

**Published:** 2021-10-09

**Authors:** Manoel E. Lixandrão, Igor Longobardi, Alice E. Leitão, João V. M. Morais, Paul A. Swinton, André Y. Aihara, Paola C. K. Goes, Carlos Ugrinowitsch, Darren G. Candow, Bruno Gualano, Hamilton Roschel

**Affiliations:** 1 Applied Physiology and Nutrition Research Group, School of Physical Education and Sport, Rheumatology Division, Faculdade de Medicina FMUSP, University of São Paulo, São Paulo 01246-903, SP, Brazil; manoel.lixandrao@gmail.com (M.E.L.); i.long@usp.br (I.L.); aliceerwig@gmail.com (A.E.L.); jvmorais1997@gmail.com (J.V.M.M.); gualano@usp.br (B.G.); 2 School of Physical Education and Sport, University of São Paulo, São Paulo 05508-030, SP, Brazil; ugrinowi@usp.br; 3 School of Health Sciences, Robert Gordon University, Aberdeen AB10 7QE, UK; p.swinton@rgu.ac.uk; 4 Laboratório Delboni Auriemo, São Paulo 04037-005, SP, Brazil; andre.yui.aihara@gmail.com (A.Y.A.); paolakuenzer@gmail.com (P.C.K.G.); 5 Faculty of Kinesiology and Health Studies, University of Regina, Regina, SK S4S 0A2, Canada; Darren.Candow@uregina.ca

**Keywords:** protein intake, aging, muscle function, essential amino acids, older individuals

## Abstract

Higher daily protein intake, with an emphasis on leucine content, is thought to mitigate age-related anabolic resistance, potentially counteracting age-related morphological and functional declines. The present study investigated potential associations between total daily leucine intake and dependent variables, including quadriceps muscle cross-sectional area (CSA) and maximum dynamic muscle strength (1-RM) in a cohort of healthy free-living older individuals of both sexes (*n* = 67; 34/33 men/women). Participants performed three 24 h dietary recalls and underwent a magnetic resonance imaging exam followed by 1-RM tests. Our results demonstrate moderate associations between total daily leucine and both quadriceps CSA (*r* = 0.42; *p* = 0.004) and 1-RM (*r* = 0.45; *p* = 0.001). Furthermore, our exploratory biphasic linear regression analyses, adjusted for sex, age, and protein intake relative to body weight, revealed a plateau for daily leucine intake and muscle mass and muscle strength (~7.6–8.0 g·day^−1^) in older adults. In conclusion, we demonstrated that total daily leucine intake is associated with muscle mass and strength in healthy older individuals and this association remains after controlling for multiple factors, including overall protein intake. Furthermore, our breakpoint analysis revealed non-linearities and a potential threshold for habitual leucine intake, which may help guide future research on the effects of chronic leucine intake in age-related muscle loss.

## 1. Introduction

Skeletal muscle mass is regulated by daily fluctuations in muscle protein synthesis (MPS) and breakdown (i.e., net protein balance). Aging, however, is known to disrupt net protein balance, blunting the MPS response to anabolic stimuli, such as exercise and protein intake (i.e., age-related anabolic resistance) [1,2,3,4]. These changes result in the loss of skeletal muscle mass [5], which is accompanied by decreases in functionality, independence, quality of life, and health span [6,7,8].

It has been proposed that a higher protein intake may help older individuals overcome age-related anabolic resistance, possibly mitigating losses in muscle mass and function [9]. Leucine content within a protein is of the utmost importance to anabolic stimulation, as it is thought to exert a pivotal role in MPS stimulation [10,11]. Devries et al. [10] demonstrated that a higher leucine concentration per meal (4.2 g) potentiated MPS, as compared with lower doses (1.3 g) in older adults, despite a similar bolus of total protein (15 g). Similarly, Murphy et al. [11] demonstrated that adding leucine to the regular meals of older individuals also increased MPS.

Despite the existing data supporting the potential benefits of higher leucine consumption during aging, international recommendations do not account for the effects of age-related anabolic resistance [12]. Nonetheless, recently, Szwiega et al. [13] estimated—using an acute infusion protocol based on an indicator amino acid oxidation technique—that the leucine requirement for older adults is more than double that of younger individuals (~78 versus 35 mg·kg^−1^·day^−1^, respectively). These results provide valuable insight, but they may be limited by a small sample size and well-controlled experimental conditions, which may not always precisely predict or correlate with neuromuscular outcomes [14,15]. In free-living conditions, dietary habits vary between individuals, providing an opportunity to use observational data to gather further insight into the possible effects of daily leucine consumption on muscle mass and function in older individuals.

Thus, this study aimed to investigate the potential impact of habitual total daily leucine intake on quadriceps muscle cross-sectional area (CSA) and maximum dynamic muscle strength (1-RM) in a cohort of healthy free-living older individuals. It was hypothesized that a cross-sectional analysis of leucine intake would demonstrate positive relationships with both morphological and functional outcomes.

## 2. Materials and Methods

### 2.1. Participants

Sixty-seven healthy elderly individuals of both sexes (34/33 men/women; age: 69.71 ± 4.04/69.36 ± 4.25 years men/women; body mass index: 26.45 ± 3.34/ 26.70 ± 4.57 kg·m^2−1^ men/women; quadriceps muscle cross-sectional area: 53.89 ± 13.00 cm^2^; unilateral maximum dynamic muscle strength: 46.38 ± 19.37 kg) participated in the study. Out of the entire sample, 24.32% presented with controlled hypertension (i.e., blood pressure within limits (<140/90 mm Hg) via medication) and 20.27% presented with controlled dyslipidemia (i.e., fasting lipids within limits (cholesterol: <200 mg·dl^−1^; triglycerides: <150 mg·dl^−1^; LDL: 130 mg·dl^−1^ and/or; HDL > 35 mg·dl^−1^) via medication). Participants’ dietary intakes are presented in Table 1. Participants were not engaged in any kind of regular resistance training and/or aerobic training for at least six months prior to the experimental period. All participants provided their informed consent before enrolling in the study. The experimental protocol was approved by the local University’s Research Ethics Committee (CAEE: 62590416.0.0000.5391).

### 2.2. Dietary Assessment

Participants performed three 24 h dietary recalls (2 non-consecutive weekdays and 1 weekend day) to determine habitual daily protein/leucine intake, using the USDA Automated Multiple-Pass Method [16]. A trained nutritionist gave oral and written instructions on how to perform the dietary recalls, including timing and estimation of portion size using household measures on an individual basis [17,18]. Upon retrieval, the same nutritionist checked the dietary recall using a visual album with real photos of foods to confirm the accuracy of the estimated portions by participants [19]. Data were analyzed with specific software (Nutritionist Pro version 7.3, Axxya Systems, Woodinville, WO, USA). Variables of interest were extracted from the software and are expressed either as total grams per day (g·day^−1^) or total grams normalized to kilograms of body mass per day (g·kg^−1^·d^−1^) (Table 1).

### 2.3. Quadriceps Muscle Cross-Sectional Area (CSA)

The quadriceps muscle cross-sectional area (CSA) of both legs was obtained through magnetic resonance imaging (MRI) (Sigma LX 9.1, GE Healthcare, Milwaukee, WI, USA). The image was acquired at 50% of the segment length (middle point between the greater trochanter to the inferior border of the lateral epicondyle of the femur) in 8 mm slices for 3 s. The pulse sequence was performed with a view field between 400 and 420 mm, a time repetition of 350 ms, an echo time from 9 to 11 ms, two signal acquisitions, and a matrix reconstruction of 256 × 256. The quadriceps CSA was measured by computerized planimetry (i.e., the region of interest was contoured following the muscle fascia) (ImageJ, version 1.53c, National Institutes of Health, Bethesda, MD, USA). The within-researcher measurement error was < 1% (i.e., same MRI image measurement with 72 h interval) [20].

### 2.4. Unilateral Maximum Dynamic Muscle Strength

The unilateral maximum dynamic muscle strength of both legs was assessed using the 1-RM test on a leg-extension machine (Movement technology, Burden, Sao Paulo, Brazil) following previous recommendations [21]. Upon the first visit, participants were familiarized with the 1-RM test. After 48 h, participants performed two 1-RM test sessions interspaced by 72 h. If any participant had a difference higher than 5% between 1-RM tests, an additional session was performed. The protocol consisted of a 5 min general warm-up on a cycle ergometer (Movement technology, Burden, Sao Paulo, Brazil). This procedure was followed by a specific warm-up with submaximal loads (i.e., 8 repetitions at 50% and 3 repetitions at 80% of the estimated 1-RM). The rest interval between warm-up sets was 1 min. After a 3 min resting period, participants had up to five attempts to achieve their 1-RM, and a 3 min rest interval was allowed between attempts.

### 2.5. Statistical Analyses

Correlation (Pearson coefficient) and regression-based analyses were conducted to quantify the relationships between total daily leucine intake (g·day^−1^) and two dependent variables, including a morphological (average bilateral quadriceps CSA) and functional (average bilateral 1-RM) outcome. Based on previous research suggesting non-linear relationships between protein/leucine content and outcomes [9,13], both linear regression and biphasic linear regression including a breakpoint (upper threshold with the second slope set to zero) were included. To control for potential confounding, the regression models also included sex, age, and total daily protein normalized to body weight (g·kg^−1^·d^−1^). Pearson’s correlation coefficients were calculated between independent and dependent variables and between each independent variable, ensuring all values were below 0.7 (analysis identified *r* < |0.65|) to reduce the potential for multicollinearity. Model comparison (biphasic versus simple linear regression) was completed via a likelihood ratio test and the appropriate standard chi-squared asymptotic reference distribution. An a priori alpha of 0.05 was used as a decision rule to define compatibility/incompatibility between each hypothesis and the data (given the model used to generate each *p* value). Analyses were performed in R statistical software (Version 3.6.3, R Foundation for Statistical Computing, Vienna, Austria) and Rstudio (Version 1.4.1103, Rstudio, Inc., Boston, MA, USA). Data are presented as mean ± standard deviation, unless otherwise stated.

## 3. Results

**Figure 1:**
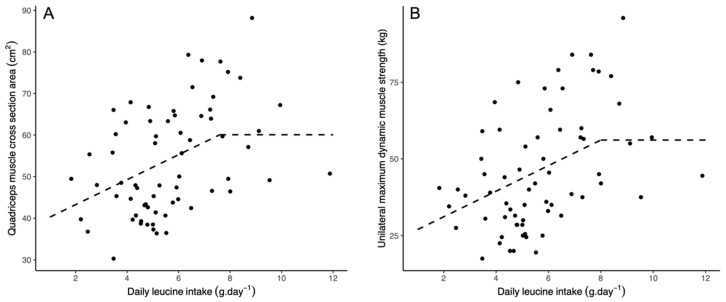
Linear biphasic regression models. (**A**): Relationship between total daily leucine intake and quadriceps muscle cross-sectional area. (**B**): Relationship between total daily leucine intake and unilateral maximum dynamic muscle strength.

There was a moderate and positive association between total daily leucine intake (g·day^−1^) and both quadriceps CSA (*r* = 0.42; *p* < 0.001) and 1-RM (*r* = 0.45; *p* < 0.001). Accounting for sex, age, and relative protein intake increased the uncertainty in the association between total daily leucine intake and both quadriceps CSA (β = 1.7, *p* = 0.051) and 1-RM (β = 2.4, *p* = 0.053). Significant improvements in overall model fit were obtained with biphasic linear regression for both quadriceps CSA (χ^2^ = 4.7, *p* = 0.030) and 1-RM (χ^2^ = 5.1, *p* = 0.023). For quadriceps CSA, a significant and positive association was obtained with total daily leucine intake (β = 3.0, *p* = 0.003), with an estimated breakpoint of 7.6 g·day^−1^ (_95%_CI: 6.5–8.7; Figure 1). Similarly, a significant positive association was obtained with 1-RM and total daily leucine intake (β = 4.2, *p* = 0.005), with an estimated breakpoint of 8.0 g·day^−1^ (_95%_CI: 6.9–9.1; Figure 1).

## 4. Discussion

The present study employed a cross-sectional design to investigate the relationship between total daily leucine intake and morphological (quadriceps CSA) and functional (1-RM) muscular parameters in free-living healthy sedentary older individuals. Our results demonstrated moderate positive correlations between total daily leucine intake and both quadriceps CSA and 1-RM. These results may be of clinical relevance, as lower-body muscle groups seem to be more negatively affected by the biological process of aging [22], and higher habitual leucine intake may serve as a potential countermeasure to these age-related decrements. Furthermore, our exploratory biphasic linear regression analysis, adjusted for confounding factors, revealed non-linearities and a potential plateau for daily leucine intake and muscle mass and muscle strength (~7.6–8.0 g·day^−1^) in older adults.

Age-related anabolic resistance to protein intake seems to be mediated by the increased splanchnic retention of proteins [23,24], as well as a reduced blood flow to musculature [25], reducing the availability of amino acids (AAs) into muscle cells. Deprivation of AAs, namely leucine, is thought to impair the phosphorylation of the mechanistic target of rapamycin complex 1 (mTORC1)—a pivotal pathway linked to anabolic responses (i.e., increases in MPS). The impaired anabolic response to feeding partially accounts for losses in skeletal muscle mass and strength during aging [5]. However, higher protein intake [9], with increased amounts of leucine [26,27], seems to mitigate this phenomenon. Acute and short-term trials (up to 2 weeks) with leucine supplementation (3–4 g per meal) in older adults have shown robust increases in MPS [11,27,28,29]; thus, suggesting that leucine could be used as a non-pharmacological strategy to counteract muscle wasting and functional declines during aging.

Based on appealing acute findings of leucine supplementation, observational data analyses have investigated the protective effects of habitual leucine intake on morphological and functional outcomes during aging [30,31]. Chae et al. [30] observed a positive and significant correlation between leucine intake and muscle mass index in healthy middle-aged non-obese individuals (50–64 years). Similar results were observed in an older cohort (>65 years), with a significant correlation between leucine intake and handgrip strength [31]. Besides corroborating with previous reports, our results expand the literature by showing the importance of leucine intake for muscle mass in individuals above 65 years. It could be speculated that consuming higher amounts of leucine daily increases its availability within the muscle, potentiating MPS in older individuals. Altogether, these results reinforce the notion that adequate leucine intake may protect against age-related muscle wasting and its concomitant functional declines.

Despite this, leucine recommendations specifically tailored for older adults are still scarce. To shed further light on the topic, we used a breakpoint approach in our retrospective dataset using individuals in free-living conditions. Our results yield an estimated breakpoint of 7.6 g·day^−1^ of leucine for muscle mass. In addition, our results expand on previous findings, as we also observed a breakpoint for lower limb muscle strength (8.0 g·day^−1^ of leucine). These data indicate that even in non-laboratory-based experimental conditions, the association between habitual leucine intake and morphological and functional outcomes follows a somewhat daily saturable dose–response pattern. In a related study, Szwiega et al. [13] estimated, using the indicator amino acid oxidation (IAAO) method, that older adults’ requirements for leucine may be higher than the current recommendation (~78 mg·kg^−1^·day^−1^ vs. 34–39 mg·kg^−1^·day^−1^). However, this estimation does not directly relate to the amount of leucine needed to maximally stimulate the rates of MPS. This notion is corroborated by the higher value for the breakpoint of leucine intake observed in our study (7.6–8.0 g·day^−1^, which is equivalent to ~110–115 mg·kg^−1^·day^−1^), and those observed by acute studies investigating the impact of additional leucine intake on integrated 3-day rates of MPS (~20–23 g·day^−1^, which is equivalent to ~240–280 mg·kg^−1^·day^−1^) [11]. Collectively, data from Szwiega et al. [13] and Murphy et al. [11], and our exploratory data analysis suggest potential cutoffs that can be used to titrate leucine requirements for older adults targeting muscle mass preservation and serve as guidance for future clinical trials aiming to test these values in a clinical setting. 

This study is not without limitations. We estimated total daily leucine intake based on dietary recalls, which, despite our robust control of the method, may be prone to over- or under-reporting dietary intake. The sample size may be considered small for observational data analyses; however, based on the significant correlations observed herein, we posit that our results hold clinical value. Finally, our study design precludes inferring cause–effect relationships. Previous prospective randomized controlled trials have failed to demonstrate the effects of leucine supplementation associated [32,33] or not [34,35] with exercise in promoting gains in muscle mass or mitigating age-related muscle loss. Notably, none of these studies provided additional protein in their dietary intervention, which has proven to be critical in overcoming the anabolic resistance of aging. This suggests that, in the clinical setting, supplementary leucine alone (without increasing total protein intake) may not be sufficient to elicit a positive net protein balance, especially in more vulnerable individuals, such as the pre-frail or frail elderly [32]. Given that muscle wasting is a slow, degenerative process, longer-term trials are warranted to validate the potential beneficial role of leucine, via diet or supplementation, in aging individuals.

## 5. Conclusions

We demonstrated that total daily leucine intake is associated with muscle mass and strength in healthy older individuals. Furthermore, our breakpoint analysis revealed non-linearities and a potential threshold for habitual leucine intake, which may help guide clinical nutritional practice aimed at mitigating the morphological and functional declines associated with aging. Randomized controlled trials should test the utility of additive leucine to counteract frailty in the elderly.

## Figures and Tables

**Table 1:** Participants’ daily dietary intakes.

	Energy (kcal·day^−1^)	Protein (g·day^−1^)	Protein (g·kg^−1^·day^−1^)	Leucine (mg·day^−1^)	Leucine (mg·kg^−1^·day^−1^)	CHO (g·day^−1^)	Fat (g·day^−1^)
Men	2192.1 ± 660.7	87.3 ± 22.5	1.1 ± 0.3	6650.2 ± 1928.4	86.2 ± 24.1	283.4 ± 104.9	75.6 ± 27.6
Women	1670.0 ± 516.2	67.35 ± 24.0	1.0 ± 0.4	4356.7 ± 1457.6	72.3 ± 23.6	217.3 ± 64.0	57.8 ± 24.3
Total	1938.2 ± 641.2	77.5 ± 25.0	1.1 ± 0.3	5666.9 ± 1975.1	79.4 ± 24.7	251.7 ± 89.8	67.6 ± 27.6

Data are presented as mean standard deviation; g: grams; mg: milligrams; kg: kilograms; CHO: carbohydrates.

## References

[1] Cuthbertson D., Smith K., Babraj J., Leese G., Waddell T., Atherton P., Wackerhage H., Taylor P.M., Rennie M.J. (2005). Anabolic signaling deficits underlie amino acid resistance of wasting, aging muscle. FASEB J..

[2] Fry C.S., Drummond M.J., Glynn E.L., Dickinson J.M., Gundermann D.M., Timmerman K.L., Walker D.K., Dhanani S., Volpi E., Rasmussen B.B. (2011). Aging impairs contraction-induced human skeletal muscle mTORC1 signaling and protein synthesis. Skelet. Muscle.

[3] Kumar V., Selby A., Rankin D., Patel R., Atherton P., Hildebrandt W., Williams J., Smith K., Seynnes O., Hiscock N. (2009). Age-related differences in the dose-response relationship of muscle protein synthesis to resistance exercise in young and old men. J. Physiol..

[4] Wall B.T., Gorissen S.H., Pennings B., Koopman R., Groen B.B., Verdijk L.B., van Loon L.J. (2015). Aging Is Accompanied by a Blunted Muscle Protein Synthetic Response to Protein Ingestion. PLoS ONE.

[5] Rong S., Wang L., Peng Z., Liao Y., Li D., Yang X., Nuessler A.K., Liu L., Bao W., Yang W. (2020). The mechanisms and treatments for sarcopenia: Could exosomes be a perspective research strategy in the future?. J. Cachexia Sarcopenia Muscle.

[6] Hunter G.R., McCarthy J.P., Bamman M.M. (2004). Effects of resistance training on older adults. Sports Med..

[7] Lipsitz L.A., Nakajima I., Gagnon M., Hirayama T., Connelly C.M., Izumo H., Hirayama T. (1994). Muscle strength and fall rates among residents of Japanese and American nursing homes: An International Cross-Cultural Study. J. Am. Geriatr. Soc..

[8] Malmstrom T.K., Miller D.K., Simonsick E.M., Ferrucci L., Morley J.E. (2016). SARC-F: A symptom score to predict persons with sarcopenia at risk for poor functional outcomes. J. Cachexia Sarcopenia Muscle.

[9] Moore D.R., Churchward-Venne T.A., Witard O., Breen L., Burd N.A., Tipton K.D., Phillips S.M. (2015). Protein ingestion to stimulate myofibrillar protein synthesis requires greater relative protein intakes in healthy older versus younger men. J. Gerontol. Ser. A Biol. Sci. Med. Sci..

[10] Devries M.C., McGlory C., Bolster D.R., Kamil A., Rahn M., Harkness L., Baker S.K., Phillips S.M. (2018). Protein leucine content is a determinant of shorter- and longer-term muscle protein synthetic responses at rest and following resistance exercise in healthy older women: A randomized, controlled trial. Am. J. Clin. Nutr..

[11] Murphy C.H., Saddler N.I., Devries M.C., McGlory C., Baker S.K., Phillips S.M. (2016). Leucine supplementation enhances integrative myofibrillar protein synthesis in free-living older men consuming lower- and higher-protein diets: A parallel-group crossover study. Am. J. Clin. Nutr..

[12] Joint Whofaounuec (2007). Protein and Amino Acid Requirements in Human Nutrition.

[13] Szwiega S., Pencharz P.B., Rafii M., Lebarron M., Chang J., Ball R.O., Kong D., Xu L., Elango R., Courtney-Martin G. (2021). Dietary leucine requirement of older men and women is higher than current recommendations. Am. J. Clin. Nutr..

[14] Mayhew D.L., Kim J.S., Cross J.M., Ferrando A.A., Bamman M.M. (2009). Translational signaling responses preceding resistance training-mediated myofiber hypertrophy in young and old humans. J. Appl. Physiol..

[15] Mitchell C.J., Churchward-Venne T.A., Parise G., Bellamy L., Baker S.K., Smith K., Atherton P.J., Phillips S.M. (2014). Acute post-exercise myofibrillar protein synthesis is not correlated with resistance training-induced muscle hypertrophy in young men. PLoS ONE.

[16] Moshfegh A.J., Rhodes D.G., Baer D.J., Murayi T., Clemens J.C., Rumpler W.V., Paul D.R., Sebastian R.S., Kuczynski K.J., Ingwersen L.A. (2008). The US Department of Agriculture Automated Multiple-Pass Method reduces bias in the collection of energy intakes. Am. J. Clin. Nutr..

[17] Polacow V.O., Scagliusi F.B., Lancha Junior A.H. Qualitative evaluation of an auxiliary tool for portion sizes estimation among a brazilian sample. Proceedings of the International Conference on Dietary Assessment Methods—Expanding the Horizon: Dietary Assessment in a Multi-Cultural World.

[18] Polacow V.O., Scagliusi F.B., Lancha Junior A.H. Validation of a portion-size measurement aid in a brazilian sample. Proceedings of the International Conference on Dietary Assessment Methods—Expanding the Horizon: Dietary Assessment in a Multi-Cultural World.

[19] Hess M., Powers C.H. (2014). Portion Photos of Popular Foods.

[20] Lixandrao M.E., Ugrinowitsch C., Bottaro M., Chacon-Mikahil M.P., Cavaglieri C.R., Min L.L., de Souza E.O., Laurentino G.C., Libardi C.A. (2014). Vastus lateralis muscle cross-sectional area ultrasonography validity for image fitting in humans. J. Strength Cond. Res..

[21] Brown L.E., Weir J. (2001). ASEP Procedures recommendation I: Accurate assessment of muscular strength and power. J. Exercise Physiol. Online.

[22] Candow D.G., Chilibeck P.D. (2005). Differences in size, strength.;power of upper and lower body muscle groups in young and older men. J. Gerontol. A Biol. Sci. Med. Sci..

[23] Boirie Y., Gachon P., Beaufrere B. (1997). Splanchnic and whole-body leucine kinetics in young and elderly men. Am. J. Clin. Nutr..

[24] Volpi E., Mittendorfer B., Wolf S.E., Wolfe R.R. (1999). Oral amino acids stimulate muscle protein anabolism in the elderly despite higher first-pass splanchnic extraction. Am. J. Physiol..

[25] Timmerman K.L., Lee J.L., Fujita S., Dhanani S., Dreyer H.C., Fry C.S., Drummond M.J., Sheffield-Moore M., Rasmussen B.B., Volpi E. (2010). Pharmacological vasodilation improves insulin-stimulated muscle protein anabolism but not glucose utilization in older adults. Diabetes.

[26] Devries M.C., McGlory C., Bolster D.R., Kamil A., Rahn M., Harkness L., Baker S.K., Phillips S.M. (2018). Leucine, Not Total Protein, Content of a Supplement Is the Primary Determinant of Muscle Protein Anabolic Responses in Healthy Older Women. J. Nutr..

[27] Katsanos C.S., Kobayashi H., Sheffield-Moore M., Aarsland A., Wolfe R.R. (2006). A high proportion of leucine is required for optimal stimulation of the rate of muscle protein synthesis by essential amino acids in the elderly. Am. J. Physiol. Endocrinol. Metab..

[28] Bukhari S.S., Phillips B.E., Wilkinson D.J., Limb M.C., Rankin D., Mitchell W.K., Kobayashi H., Greenhaff P.L., Smith K., Atherton P.J. (2015). Intake of low-dose leucine-rich essential amino acids stimulates muscle anabolism equivalently to bolus whey protein in older women at rest and after exercise. Am. J. Physiol. Endocrinol. Metab..

[29] Casperson S.L., Sheffield-Moore M., Hewlings S.J., Paddon-Jones D. (2012). Leucine supplementation chronically improves muscle protein synthesis in older adults consuming the RDA for protein. Clin. Nutr..

[30] Chae M., Park H.S., Park K. (2021). Association between dietary branched-chain amino acid intake and skeletal muscle mass index among Korean adults: Interaction with obesity. Nutr. Res. Pr..

[31] Park S., Chae M., Park H., Park K. (2021). Higher Branched-Chain Amino Acid Intake Is Associated with Handgrip Strength among Korean Older Adults. Nutrients.

[32] Roschel H., Hayashi A.P., Fernandes A.L., Jambassi-Filho J.C., Hevia-Larrain V., de Capitani M., Santana D.A., Goncalves L.S., de Sa-Pinto A.L., Lima F.R. (2021). Supplement-based nutritional strategies to tackle frailty: A multifactorial, double-blind, randomized placebo-controlled trial. Clin. Nutr..

[33] Trabal J., Forga M., Leyes P., Torres F., Rubio J., Prieto E., Farran-Codina A. (2015). Effects of free leucine supplementation and resistance training on muscle strength and functional status in older adults: A randomized controlled trial. Clin. Interv. Aging.

[34] Leenders M., Verdijk L.B., van der Hoeven L., van Kranenburg J., Hartgens F., Wodzig W.K., Saris W.H., van Loon L.J. (2011). Prolonged leucine supplementation does not augment muscle mass or affect glycemic control in elderly type 2 diabetic men. J. Nutr..

[35] Verhoeven S., Vanschoonbeek K., Verdijk L.B., Koopman R., Wodzig W.K., Dendale P., van Loon L.J. (2009). Long-term leucine supplementation does not increase muscle mass or strength in healthy elderly men. Am. J. Clin. Nutr..

